# Rapidly responsive silk fibroin hydrogels as an artificial matrix for the programmed tumor cells death

**DOI:** 10.1371/journal.pone.0194441

**Published:** 2018-04-04

**Authors:** Viviana P. Ribeiro, Joana Silva-Correia, Cristiana Gonçalves, Sandra Pina, Hajer Radhouani, Toni Montonen, Jari Hyttinen, Anirban Roy, Ana L. Oliveira, Rui L. Reis, Joaquim M. Oliveira

**Affiliations:** 1 3B’s Research Group – Biomaterials, Biodegradables and Biomimetics, University of Minho, Headquarters of the European Institute of Excellence on Tissue Engineering and Regenerative Medicine, Avepark – Parque de Ciência e Tecnologia, Zona Industrial da Gandra, Barco, Guimarães, Portugal; 2 ICVS/3B's – PT Government Associated Laboratory, Braga/Guimarães, Portugal; 3 Computational Biophysics and Imaging Group, ELT Department, Tampere University of Technology, Tampere, Finland; 4 BioMediTech - Institute of Biosciences and Medical Technology, Tampere, Finland; 5 Anasys Instruments Corp - Santa Barbara, California, United States of America; 6 CBQF – Centro de Biotecnologia e Química Fina - Laboratório Associado, Escola Superior de Biotecnologia, Universidade Católica Portuguesa, Porto, Portugal; 7 The Discoveries Centre for Regenerative and Precision Medicine, Headquarters at University of Minho, Avepark – Parque de Ciência e Tecnologia, Zona Industrial da Gandra, Barco, Guimarães, Portugal; University of South Carolina, UNITED STATES

## Abstract

Timely and spatially-regulated injectable hydrogels, able to suppress growing tumors in response to conformational transitions of proteins, are of great interest in cancer research and treatment. Herein, we report rapidly responsive silk fibroin (SF) hydrogels formed by a horseradish peroxidase (HRP) crosslinking reaction at physiological conditions, and demonstrate their use as an artificial biomimetic three-dimensional (3D) matrix. The proposed SF hydrogels presented a viscoelastic nature of injectable hydrogels and spontaneous conformational changes from random coil to β-sheet conformation under physiological conditions. A human neuronal glioblastoma (U251) cell line was used for screening cell encapsulation and *in vitro* evaluation within the SF hydrogels. The transparent random coil SF hydrogels promoted cell viability and proliferation up to 10 days of culturing, while the crystalline SF hydrogels converted into β-sheet structure induced the formation of TUNEL-positive apoptotic cells. Therefore, this work provides a powerful tool for the investigation of the microenvironment on the programed tumor cells death, by using rapidly responsive SF hydrogels as 3D *in vitro* tumor models.

## Introduction

Hydrogels are hydrophilic networks with high capacity to absorb and retain high quantities of water, while keeping its original structure [1]. Smart hydrogels, or stimuli-responsive hydrogels, are more appealing for cell encapsulation in a three-dimensional (3D) microenvironment, drug delivery systems and tissue engineering (TE) scaffolding. In fact, the possibility to creating such smart hydrogels capable of harboring cell ingrowth/organization and at the same time promoting the delivery of biologically active molecules due to the rapid response to environmental stimuli and high elasticity, was a great achievement in the biomedical field [2]. In the last years, several physical and chemical crosslinking methods have been developed to produce artificial hydrogel matrices temporally and spatially regulated [3]. The production of injectable hydrogels for minimally invasive clinical applications has been receiving special attention [4]. The *in situ* formation of these hydrogels is based on the aqueous mixture of polymer solutions with bioactive agents that when injected into the body will form a desired hydrogel shape into the defect site, even oddly shaped ones. The precursor hydrogel solutions can also be combined with cells, drugs and growth factors, and subsequently injected into the application site [5]. Recently, an increasing interest has been devoted to enzymatically crosslinked hydrogels to be applied as injectable matrices for the generation of functional tissue substitutes able to maintain cell phenotype of the native tissue, which is highly important for tissues like cartilage [6]. These *in situ* forming hydrogels are produced through mild reactions that in many cases are catalyzed by enzymes naturally existing in our body [7]. The specificity of these substrates avoids toxic effects that are often observed on organic solvents or photo-initiators mediated reactions [8]. Another major advantage of the enzymatic crosslinking reactions is that they can be easily applied with natural polymers that hardly support more adverse chemical or physical reactions. Moreover, the polymerization reaction can be controlled by modulating the enzyme activity, affecting the gelation rate and the hydrogels mechanical properties [8, 9].

Hydrogels derived from natural polymers have been attracting special attention since they can more closely simulate the natural extracellular matrix (ECM) environment. Some of the most studied natural hydrogels include alginate, fibrin, collagens, gelatin, chitosan and hyaluronic acid [10]. Silk fibroin (SF) derived from *Bombyx mori* silkworm is a biodegradable and biocompatible natural material that has been extensively studied for TE applications using different forms, including as membranes [11], films [12], fibres [13, 14], sponges [15] and textiles [16]. SF hydrogels have been developed by the protein conformation transition from amorphous to β-sheet induced by physical or chemical crosslinking methods involving external stimuli and long gelation times [17, 18]. In this sense, an enzyme mediated crosslinking may be the ideal method to produce fast-gelled SF-based injectable hydrogels at physiologic conditions. Our group proposed SF hydrogels preparation via a horseradish peroxidase (HRP)-mediated crosslinking in physiological conditions [19–21]. In their first systematic study, the authors observed that varied concentrations of SF and HRP/hydrogen peroxide (H_2_O_2_) crosslinking solutions lead to different physicochemical properties of the SF hydrogels [19]. These enzymatic crosslinking approach has shown great potential for preparing injectable hydrogels from polymers containing or functionalized with phenol group-containing molecules, including tyrosine, tyramine or aminophenol [8]. Considering that SF contains 5.3% tyrosine molecules with the required phenol groups [22], such approach was explored to produce *in situ* fast-formed hydrogels (S1 Fig) [23–25]. These SF hydrogels can exhibit a spontaneous conformation change under physiological conditions. Therefore, understand if and how the protein conformation changes affect cell behavior and tissue ingrowth was another major concern in applying this enzymatic crosslinking mechanism.

The 3D cell culture plays an important role in tumor biology since it enables to create an *in vivo* like microenvironment. Among the existing tools of experimental cancer research, spheroids represent an advanced *in vitro* model compared to the standard 2D cell culture, and less invasive as compared to the animal tumor models [26]. At the same time, the 3D spheroids can be expensive, time consuming and hard to obtain in more complex tumor-like model approaches [27]. Recently, cancer cell culture on 3D platforms have been attracting much attention. The use of 3D matrices has shown to closely mimic the natural tumor ECM, allowing cells to grow and show similar properties to those of cells growing under physiological conditions. In the literature, biomimetic scaffolds made of branched hollow silica microfibers were proposed to culture cancer cells in a 3D environment [28], showing that the proposed scaffolds mimic the fibrous ECM of real tumors and allowed cells to form tumor-like multicellular spheroids *in vitro* and promote tumor growth *in vivo*. Macroporous polymeric scaffolds of nanofibrous bacterial cellulose have also been successfully proposed as *in vitro* models for the culture of breast cancer cells [29].

Among the different 3D platforms established for cancer therapy studies, hydrogels demonstrate some peculiarities in terms of response to therapeutic agents [30, 31]. For example, microfluidic 3D hydrogel models were proposed to assess anti-cancer drugs interactions for bone cancer research and therapy [32]. Previous studies, have also shown that hydrogels can be effective in studies of tumor-host interactions that regulate tumor formation and progression [33, 34]. Using a different approach, SF-based hydrogels were proposed for the controlled delivery of plasmid DNA for specific cancer gene therapy applications [35]. Another reason that promotes the use of hydrogel systems for cancer research is because they can be applied in a minimally invasive manner and over a wide range of temporal profiles. The injectable *in situ* crosslinked hydrogels ensure the localized and painless administration of cytotoxic drugs that are usually applied systemically in conventional chemotherapy, producing generalized side effects [36]. For example, in different studies anticancer-loaded injectable hydrogels have been tested as platform for the local delivering of chemotherapeutic drugs after glioblastoma resection, that is the most common, aggressive and inaccessible primary brain tumor in adults [37]. Considering the suitable properties of SF as a natural polymer platform and its applicability for producing injectable and non-toxic hydrogels, this would be an advantageous model system for cancer therapy applications.

Herein, we deeply investigate the conformational transitions of rapidly responsive SF hydrogels from random coil to β-sheet and its potential use as a biomimetic matrix for the programmed tumor cells death in 3D *in vitro* tumor models. An enzyme-mediated crosslinking method using HRP and H_2_O_2_ was used to produce in a few minutes *in situ* formed SF hydrogels at physiological conditions [20] (S1 and S2a Figs). The spontaneous β-sheet conformation transition on the protein-based hydrogels was morphological and chemically investigated at multi-scale, and the rheological properties determined. We further explored the *in vitro* cell encapsulation within the proposed hydrogels. A human neuronal glioblastoma (U251) cell line was used to evaluate how the cells respond to the protein conformational changes, up to 14 days of culturing (S2b and S2c Fig).

## Material and methods

### Materials

Silk derived from the silkworm *Bombyx mori* in the form of coccons was provided by the Portuguese Association of Parents and Friends of Mentally Disabled Citizens (APPACDM, Castelo Branco, Portugal). All reagents were purchased from Sigma-Aldrich (St. Louis, MO, USA) unless otherwise stated.

### Preparation of silk fibroin aqueous solution and hydrogels

Purified silk fibroin (SF) was prepared by removing the glue-like protein sericine from the cocoons in a 0.02 M boiling sodium carbonate solution for 1 hour, followed by rising with distilled water in order to fully remove the degumming solution [16]. A 9.3 M lithium bromide solution was used to dissolve the purified SF for 1 hour at 70°C and dialyzed in distilled water for 48 hours using the benzoylated dialysis tubing (MWCO: 2 kDa). In the last 12 hours, SF was dialyzed in phosphate buffer saline solution (PBS, without calcium or magnesium ions) and then concentrated against a 20 wt.% poly(ethylene glycol) solution for at least 6 hours. The final concentration of SF was determined by measuring the dry weight of the SF solution placed in the oven at 70°C overnight. Meanwhile, the prepared SF solution was stored at 4°C until further use.

SF hydrogels were prepared according to the procedure described by Yan *et al*. [20]. Briefly, the stored SF solution was diluted to 16 wt.% in PBS and combined with horseradish peroxidase solution (HRP type VI, 0.84 mg/mL) and hydrogen peroxide solution (H_2_O_2_, 0.36 wt.%; Panreac, Barcelona, Spain), both also prepared in PBS. A mixture of 1 mL SF solution, 100 μL HRP solution and 65 μL H_2_O_2_ solution (1/0.52‰/1.45‰) was prepared in a 1.5 mL centrifuge tube (Eppendorf, Hamburg, Germany), and warmed in a water bath of 37°C. This formulation was chosen after some optimization process [20]. SF hydrogels were prepared by the deposition of 100 μL of mixture in tissue culture polystyrene (TCPS) coverslips (22 mm diameter, Sarstedt, Nümbrecht, Germany), unless otherwise mentioned, followed by the complete gelation in the oven at 37°C. The prepared hydrogel discs were used for further characterization tests performed after 1, 3, 7, 10 and 14 days of hydrogels formation.

### Structural analysis of the SF hydrogels

#### Transmission electron microscopy

Transmission electron microscopy (TEM) was used to evaluate the natural ability of SF to form β-sheet fibrils. SF hydrogel discs were contrasted through negative staining using 2% uranyl acetate for 5 seconds. TEM images were acquired using a JEOL JEM 1400 TEM (Tokyo, Japan) and digitally recorded using a CCD digital camera Orious 1100W Tokyo, Japan.

#### Thioflavin T staining

The qualitative evidence of the β-sheet structure on SF hydrogels was tested using the dye thioflavin T. A 1% (w/v) thioflavin T solution was used to stain the SF hydrogels for 10 minutes followed by rinse with 70% ethanol solution and then washed with distilled water three times. Stained samples were observed under transmitted and fluorescence microscopy (ex/em 495/515 nm) using a transmitted and reflected light microscope (Axio Imager Z1m, Zeiss, Jena, Gernamy). Images were acquired using the digital cameras AxioCam MRc5 or MR3 (Zeiss, Jena, Germany), respectively, connected to the Zen microscope processing software (Zeiss, Jena, Germany).

### Physicochemical characterization of the SF hydrogels

#### X-ray diffraction

The qualitative analysis of crystalline phases presented on the SF hydrogels was performed by X-ray diffraction (XRD) using a high-resolution Bragg–Brentano diffractometer (Bruker D8 Advance DaVinci, Karlsruhe, Germany) equipped with CuKα radiation (λ = 1.5418 Å), produced at 40 kV and 40 mA. SF hydrogel discs were prepared by adding 100μL of the SF/HRP/H_2_O_2_ mixture in polydimethylsiloxane (PDMS; Sylgard 184 Silicone Elastomer Kit, Dow Corning, Belgium) silicone molds (8 mm diameter and 2 mm height). Data sets were collected in the 2θ range of 10–50° with a step size of 0.02° and 1s for each step. XRD measurements were repeated three times independently.

#### Fourier transform infrared spectroscopy

The chemical composition and structural conformation of the SF hydrogels were analyzed by Fourier transform infrared (FTIR) spectroscopy (Perkin-Elmer 1600 series equipment, CA, USA) under an attenuated total reflectance (ATR) model (IRPrestige-21, Shimadzu, Japan). SF hydrogel discs were prepared as described above. All spectra were obtained between 4600 to 800 cm^-1^ at a 4 cm^-1^ resolution with 50 scans. Each specimen was examined for at least 3 times and PBS was used as background.

#### Atomic force microscopy and infrared spectroscopy

The chemical characterization of the SF hydrogels was also performed combining atomic force microscopy (AFM) and IR spectroscopy at a nanoscale spatial resolution using a Resonance enhanced mode on a NanoIR-2 system by Anasys Instruments (CA, USA), equipped with a Quantum Cascade Laser (QCL) as the IR source. Samples were prepared by spin-coating (Spin Coater Model WS-650-23, Laurell Technologies, PA, USA) ZnS sampling flat substrates (Anasys Instruments, CA, USA) with the SF/HRP/H_2_O_2_ mixture. The spectra were acquired in a 1000–1800 cm^-1^ range with a spectral resolution of 2 cm^-1^. Multiple spectra were acquired for each sample, averaged and smoothed using Savitzky-Golay filter.

#### Rheological properties

Rheological analysis was performed using a Kinexus pro+ rheometer (Malvern Instruments, UK), using the acquisition software rSpace (Malvern Instruments, UK). For the oscillatory experiments the measuring system was equipped with stainless steel (316 grade) parallel plates: an upper measurement geometry plate (8 mm diameter) and a lower pedestal (20 mm diameter) with roughened finish. Frequency sweep experiments were performed using SF hydrogel discs prepared in silicone molds (8 mm diameter and 2 mm height), as described above. The measurements were obtained by plot the frequency (Hz) as function of modulus (Pa) and with a predefined shear strain (0.53%). Temperature and time sweep experiments were performed to the SF/HRP/H_2_O_2_ mixture, using a large upper geometry plate (20 mm diameter) and the oscillatory experiments were performed at 1 Hz of frequency, 0.53% of shear strain and during 100 minutes (6000 s). The temperature sweep curve was obtained for the range of 25°C to 45°C and the time sweep curve with a fixed temperature of 37°C. For the rotational experiments the measuring system was equipped with an upper measurement geometry cone (40 mm diameter and 4° angle). Shear viscosity and shear stress were determined for the SF/HRP/H_2_O_2_ mixture, as a function of the shear rate (0.01 s^−1^ to 100 s^−1^). These experiments were performed at 37°C and all plots are the average of at least 3 samples.

### Cell culture and encapsulation in the SF hydrogels

#### U251 glioma cell line culture

Human neuronal glioblastoma (U251) cell line was generously donated by Prof. Joseph Costello (California University, Neurosurgery Department, San Francisco, USA) and further provided by Prof. Rui M. Reis (Life and Health Science Research Institute, University of Minho, ICVS/3B’s—PT Government Associate Laboratory, Portugal). All experiments and protocols related to U251 cell line were approved by the Ethics Committee of University of Minho. U251 cell line was expanded in Dulbecco’s modified Eagle’s medium (DMEM) with phenol red, supplemented with 10% fetal bovine serum (FBS; Life Technologies, Carlsbad, CA, USA) and 1% antibiotic–antimycotic (Life Technologies, Carlsbad, CA, USA). Cells were cultured until confluence in a CO_2_ incubator with an atmosphere of 5% CO_2_ at 37°C, and the culture medium was changed every 2–3 days.

#### Hydrogel encapsulation of U251 cells

A mixture of 1 mL SF solution, 100 μL HRP solution and 65 μL H_2_O_2_ solution, was warmed in a water bath (37°C) for about 6 minutes. Then, 1 mL of the warmed mixture was homogeneously mixed with a U251 cell pellet containing 1×10^6^ cells and 100 μL of cell suspension were transferred into TCPS coverslips (22 mm diameter) in a 12-well suspension cell culture plate (Corning Incorporated, Life Sciences, NC, USA), unless otherwise mentioned. The plate was then incubated for 15 minutes at 37°C in the CO_2_ incubator, to complete gelation. After the gel formation, 3 mL of basal DMEM medium were added to each well and the culture medium was changed every 2 days. Samples were collected for analysis at day 1, 7, 10 and 14.

### Cell viability and proliferation in the SF hydrogels

#### ATP bioluminescence assay

Cell viability was assessed using the CellTiter-Glo^®^ Luminescent Cell Viability Assay (Promega, WI, USA). The cell-laden hydrogels were incubated in a mixture consisting of serum-free cell culture medium and CellTiter-Glo^®^ Reagent in a 1:1 ratio, for 30 minutes at room temperature (RT). The emitted luminescence was detected in a microplate reader (Synergy HT, BioTek Instruments, Winooski, VT, USA) using a sensitivity of 120. The ATP concentration for each sample was calculated according to a standard curve prepared with concentrations ranging between 0 and 2 μmol/L, relating quantity of ATP and luminescence intensity. Hydrogels without cells were used as control.

#### DNA quantification

Cell proliferation was evaluated using the Quant-iT^®^ Pico-Green dsDNA Assay Kit (Life Technologies, Carlsbad, CA, USA), according to the manufacturer's instructions. The cell-laden hydrogels were incubated at 70°C for 30 minutes and kept in 1 mL of ultrapure water at −80°C until further analysis. Before analysis, samples were thawed at RT and sonicated for 1 hour at 37°C to induce complete membrane lysis. Supernatant fluorescence was measured in a microplate reader (ex/em 485/528 nm). The DNA concentration for each hydrogel was calculated using a standard curve with concentrations ranging from 0 to 2 μg/mL, relating quantity of DNA and fluorescence intensity. Hydrogels without cells were used as control.

#### Live/Dead staining

A Calcein AM and propidium iodide (PI; Life Technologies, Carlsbad, CA, USA) staining was performed to confirm the viability of the encapsulated cancer cells. The cell-laden hydrogels were incubated in 1 mL PBS supplemented with 1 μg Calcein AM and 5 μg PI, for 10 minutes at 37°C in the CO_2_ incubator. Samples were washed with PBS and observed under fluorescence microscopy (Calcein AM (green): ex/em 495/515 nm; PI (red): ex/em 495/635 nm) in a transmitted and reflected light microscope. Images were acquired using the digital camera MR3 connected to the respective Zen microscope software.

#### TUNEL assay

An *in situ* Cell Death Detection Kit, Fluorescein (Roche, Basel, Switzerland) was used in cell-laden hydrogels and sections from the cell-laden hydrogels (3.5 μm thick) to detect apoptotic cells, based on Terminal deoxynucleotidyl transferase dUTP nick end labeling (TUNEL) reaction and according to the manufacturer's instructions. Cell-laden hydrogels were fixed in 10% formalin for 1 hour at RT and permeabilized with 0.1% (v/v) of Triton X-100/0.1% (w/v) of sodium citrate (Fisher Scientific, NJ, USA). The sections from the cell-laden hydrogels were after paraffin removal, rehydrated and submitted to heat-induced antigen retrievel using 10 mM citrate buffer (pH 6; Panreac, Barcelona, Spain). After washed with PBS, the TUNEL reaction mixture (50 μL/sample) was added to the hydrogel samples and incubated for 1 hour at 37°C, in the dark. Negative control (without terminal transferase) and positive control (with DNase I recombinant 20 U/mL (Amresco) in 50 mM Tris(hydroxymethyl) aminomethane, pH 7.5, and 1 mg/mL bovine serum albumin) samples were also prepared. A counterstaining was performed using 4,6-Diamidino-2-phenyindole, dilactate (DAPI; Biotium, CA, USA). Samples were washed with PBS and observed under fluorescence microscopy (Apoptotic cells (green): ex/em 495/515 nm; DAPI (blue): ex/em 358/461 nm) as described above.

#### Optical projection tomography

Optical projection tomography (OPT) system was used to analyze the 3D microstructure of the SF hydrogels and to evaluate the distribution of the cell-laden SF hydrogels. SF hydrogel samples were prepared by filling fluorinated ethylene propylene (FEP) tubes with 100μL of the un-laden or cell-laden SF/HRP/H_2_O_2_ mixture. The specimens were immersed in an index-matching liquid (distilled water) and rotated through a series of angular positions. The center of rotation and the alignment of the samples were adjusted using a manual x-y-stage (Standa, Lithuania) in conjunction with the sample-positioning module (Standa, Lithuania) by using the 4 available axes (3 translational, 1 rotational). The acquisition consists of rotating a sample 360° in 0.9° steps and capturing an image at each rotation angle, ranging from 0° to 359.1°. A total of 400 images were acquired per sample. The OPT system was used in brightfield mode. The images were captured with an sCMOS camera (Hamamatsu, Japan) and the exposure time was adjusted from 4 ms to 20 ms depending on the transparency of the sample. Projections collected in each orientation were used to create the 3D reconstructions of each sample and the visualization of the 3D-volume was obtained using Avizo software (FEI, USA).

#### Selective plane illumination microscopy

Cell distribution in the SF hydrogels was also analyzed by selective plane illumination microscopy (SPIM), following the same protocol for cell-loading sample preparation described above. The samples imaged with SPIM were stained with Calcein AM (green: ex/em 494/517 nm) and Phalloidin (red: ex/em 550/575 nm). The FEP-tubes containing the samples were supported by a 4 axis-positioning device (Picard Industries, USA) and the plane of interest in each sample was found by using the 4 available axes (3 translational, 1 rotational). The fluorophore distribution in the samples was acquired my translating the sample along the detection axis while taking images at constant intervals. The images were collected with an sCMOS camera. The samples were imaged using an exposure time of 300 ms except for the day 10 and day 14 samples (exposure time of 100 ms to avoid overexposure). Images were taken in 3 μm z-steps across depth of 500 μm. Due to narrow field of view, separate stacks were acquired to cover the width of the sample. The stacks were stitched together in post processing to create a wider field of view. Finally, the stitched stacks were visualized using Avizo software.

### Statistical analysis

All the numerical results are presented as mean ± standard deviation (SD). Statistical analysis was performed using the GraphPad Prism 5.0 Software (GraphPad Software Inc., La Jolla, CA, USA). First, a Shapiro-Wilk test was used to ascertain about the data normality. The results indicated that non-parametric tests should be used for all comparisons. The comparisons were performed using the Kruskal-Wallis test followed by Dunn’s multiple comparison test. Three independent experiments were performed for all biological quantification assays, and carried out with three replicates in each culturing time. Statistical significance was set to **p < 0.01, ***p < 0.001.

## Results

### Structure and multi-scale conformational transitions of the SF hydrogels

**Fig 1:**
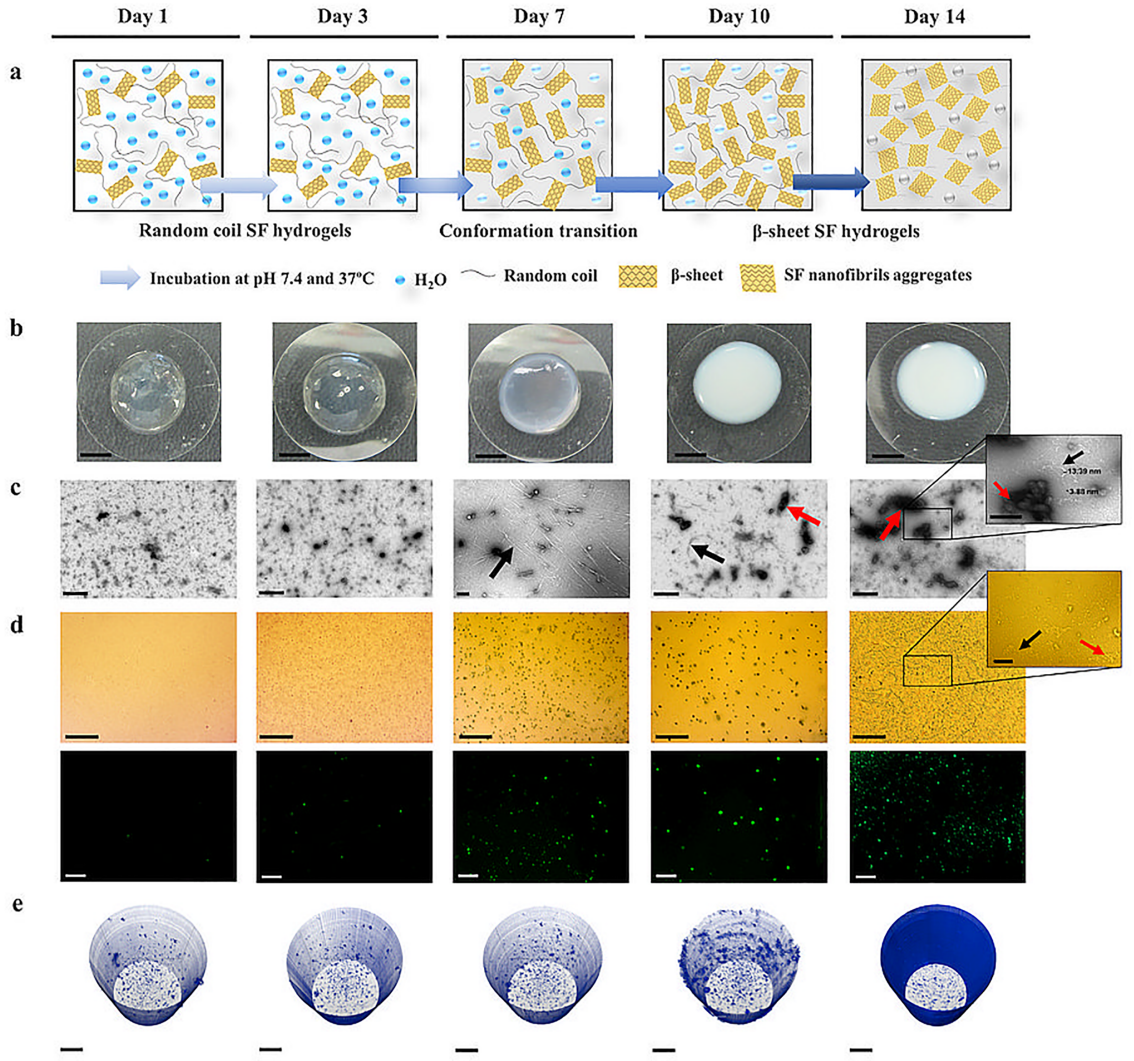
Structural evaluation of the SF hydrogels after incubation in PBS at 37°C for 1, 3, 7, 10 and 14 days. (a) Schematic illustration of the β-sheet structural transitions in the SF hydrogels. (b) Macroscopic images of the SF hydrogels (scale bar, 5 mm). (c) TEM micrographs of the SF hydrogels (scale bar, 500 nm for low magnification images; scale bar, 200 nm for high magnification image). (d) SF hydrogels labeled with thioflavin T (scale bar, 500 μm for low magnification images; scale bar, 100 μm for high magnification image) analyzed under the fluorescence microscope (scale bar, 200 μm). The black arrows indicate nanofibrils and the red arrows indicate aggregates. (e) OPT reconstructions of the SF hydrogels (scale bar, 500 μm). Movies from OPT reconstructions of the SF hydrogels are available in S1–S5 Movies.

To clarify the relationship between hydrogels morphology and conformational changes of the SF protein, we performed a multi-scale structural characterization to evaluate the conformational distribution within the SF hydrogels over the 14 days of incubation at physiological conditions (Fig 1a). SF hydrogels immersed in phosphate buffer saline solution (PBS) solution at 37°C for 14 days presented a transparent morphology over the first 7 days, becoming completely opaque after 10 days of incubation (Fig 1b) [20]. The Transmission electron microscopy (TEM) images revealed at day 1 and day 3 the presence of a high amount of small and randomly distributed SF nanofibrils, representative of a main amorphous conformation in the hydrogels (Fig 1c). At day 7, the dimensions of the SF nanofibrils increased substantially, and from day 10 to day 14 the nanofibrils aggregates also increased exponentially, indicating higher order of β-sheet structures and the typical evidence of a conversion to a dominant β-sheet conformation domain [38]. Thioflavin T (ThT) staining (Fig 1d) came to confirm the TEM observations by the increasing evidence of SF nanofibrils and aggregates over the 14 of hydrogels incubation. The bright green fluorescence detecting protein nanofibrils in the β-sheet state was only observed from day 7, confirming the previous observations [39]. In Fig 1e and S1–S5 Movies, are represented the 3D reconstructions of the SF hydrogels obtained by Optical projection tomography (OPT) analysis. The low intensity voxels colored in blue, give a clear perception of the hydrogels microstructure and highlight their conformational changes. From day 1 to day 7, hydrogels presented a homogeneous intensities distribution characteristic of transparent hydrogels (random coil main conformation). From day 10 until day 14, an increase in entropy was observed, which is related with a random intensity distribution in high density hydrogels (β-sheet main conformation) [40, 41].

**Fig 2:**
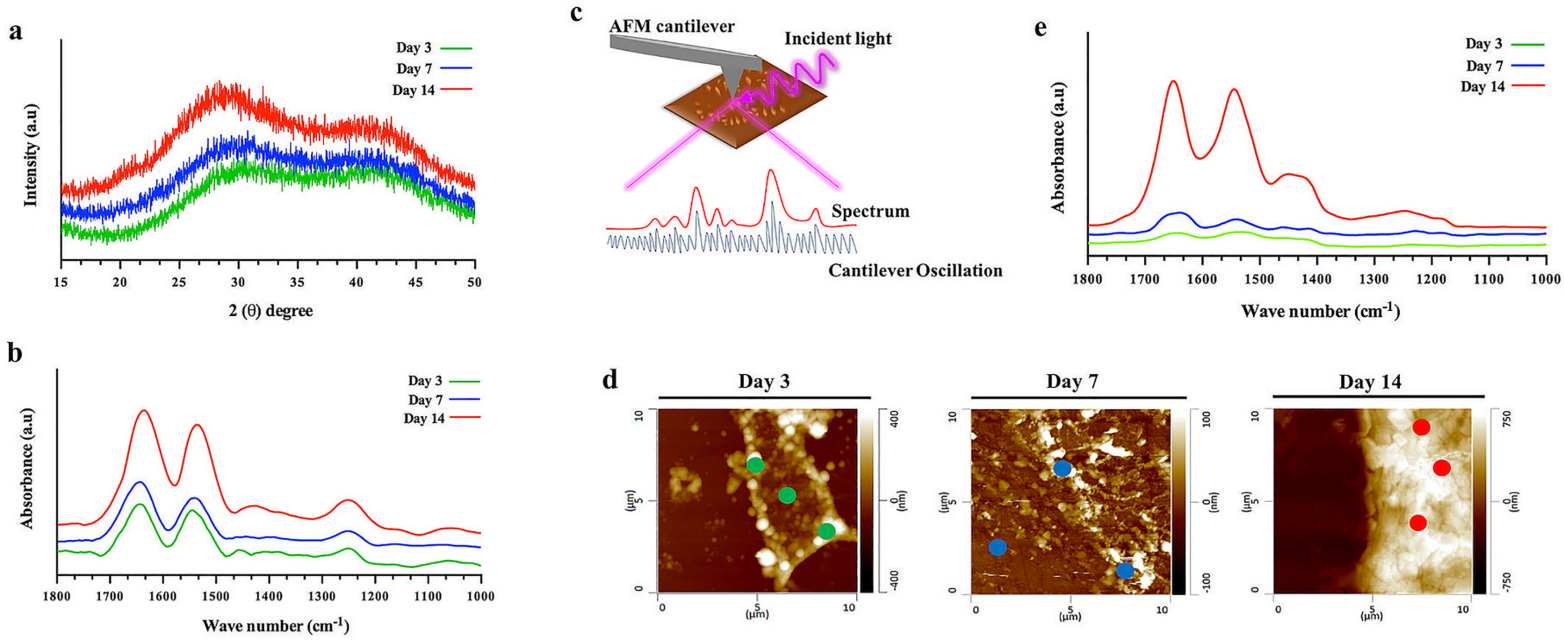
Physicochemical characterization of the SF hydrogel after incubation in PBS at 37°C for 3, 7 and 14 days. (a) XRD patterns of the SF hydrogels. (b) ATR-FTIR spectra of the SF hydrogels. (c) Schematic illustration of nanoscale IR spectroscopy using AFM-IR: IR pulses are emitted in the sample increasing the local absorption on SF nanofibrils acquired by the AFM cantilever tip, corresponding to the absorption spectroscopic peaks. (d) Tapping-mode AFM nano-images (10 μm x 10 μm), and (e) IR nano-spectra of the SF hydrogels obtained by measuring the samples at different points selected by the AFM tip, corresponding to the absorption spectroscopic signatures and indicated as green, blue and red points.

The conformational changes of SF hydrogels were then confirmed by X-ray diffraction (XRD, Fig 2a) and attenuated total reflectance Fourier transform infrared spectroscopy (ATR-FTIR, Fig 2b). XRD analysis revealed no significant differences between the hydrogels tested with different incubation periods at 37°C (3, 7 and 14 days) in terms of peak positions, ranging from 2θ = 28.4–30.8°. The intensity of these peaks increased over time, which characterizes the typical pattern of substances undergoing an increase of crystallinity [42], represented by broad peaks at the first tested periods (3 and 7 days) converted to a crystalline diffraction pattern after 14 days of incubation. The peak identified on day 14 at 2θ = 28.4° can be assigned to β-sheet crystalline domain [43] and the additional peak at 2θ = 21°, was only detected at day 14 and also characterizes the β-sheet structure [44]. The main absorbance peaks detected by ATR-FTIR for the SF hydrogels were on day 3 at 1643 cm^-1^ and 1545 cm^-1^ and on day 7 at 1643 cm^-1^ and 1541 cm^-1^. These peaks are characteristic of random coil conformation and correspond to the amide I and amide II bands, respectively [45, 46]. At day 14, strong absorption peaks were detected at 1636 cm^-1^ and 1535 cm^-1^, corresponding to the β-sheet structure on the SF hydrogels [45]. From day 3 to day 14, hydrogels presented an absorbance peak between 1250 cm^-1^ to 1252 cm^-1^, corresponding to amide III band positions. As expected, the intensity of this peak was higher at day 14, which is an indication of the higher β-sheet crystalline domain in these hydrogels [46]. We further quantitatively and unambiguously confirmed the structural transitions between the SF hydrogels at a nanoscale by atomic force microscopy combined to IR nano-spectroscopy (AFM-IR) (Fig 2c). Fig 2d, shows the AFM nano-imaging of the SF hydrogel samples at the different tested periods, revealing that after 3 and 7 days of incubation at 37°C a series of elements were exhibited, with the presence of some globular amorphous protein aggregates with different sizes and morphologies. At day 14, a large globular protein aggregate was identified with an opaque morphology, confirming that SF hydrogels underwent conformational changes [38]. The AFM-IR spectra (Fig 2e) obtained for the SF hydrogels were correlated to that obtained with the conventional ATR-FTIR (Fig 2b). Nevertheless, through AFM-IR analysis it was possible to obtain an average structural information of samples covering larger areas of analysis, which is an important advancement considering the high structural heterogeneity of SF hydrogels at the nanoscale (Fig 2d) [47]. The amide band positions (I, II, II) of samples from day 3 and day 7 were quite similar and of low intensity (indistinct peaks), characteristic of a random coil main conformation (1644 cm^-1^ and 1640 cm^-1^ for amide I, 1532 cm^-1^ and 1540 cm^-1^ for amide II and 1232 cm^-1^ and 1228 cm^-1^ for amide III, respectively) [45, 46]. At day 14, all the amide signature bands shift towards higher frequency, especially those of amide I and amide II (1650 cm^-1^ for amide I, 1546 cm^-1^ for amide II and 1256 for amide III), which is a characteristic of the β-sheet structure [48, 49]. Therefore, these results suggest that the natural conformational changes of the native SF can also spontaneously occur on the SF hydrogels over time.

### Viscoelastic properties and hydrogelation kinetics of the SF hydrogels

**Fig 3:**
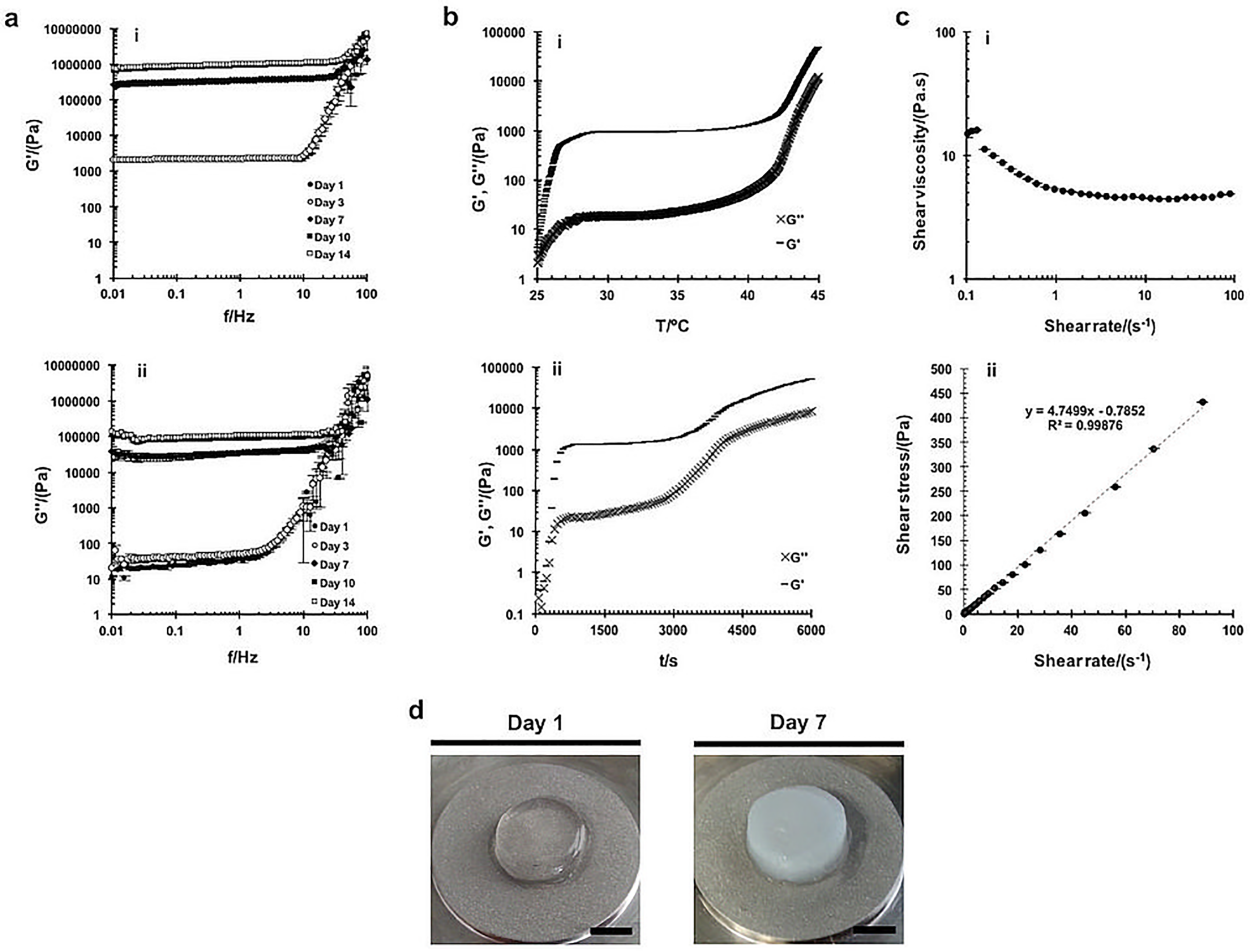
Rheological properties of the SF/HRP/H_2_O_2_ mixture before gelation, after SF hydrogels formation and incubation in PBS at 37°C for 1, 3, 7 and 14 days. (a) Oscillatory experiments or frequency sweep curves: (i) storage modulus as a function of frequency, and (ii) loss modulus as a function of frequency for the SF hydrogels. (b) Dynamic moduli as function of: (i) temperature, and (ii) time for the SF/HRP/H_2_O_2_ mixture. (c) Rotational experiments: (i) shear viscosity, and (ii) shear stress as a function of shear rate for the SF/HRP/H_2_O_2_ mixture. (d) Macroscopic images of SF hydrogels analyzed at day 1 and day 7 (scale bar, 4 mm).

The mechanical properties of the SF hydrogels and SF/HRP/H_2_O_2_ mixture before gelation were analyzed by rheometer, detecting the frequency sweep as a useful tool to characterize the microstructure of a viscoelastic material. Frequency sweep curves obtained from oscillatory shear measurements performed on the SF hydrogels are shown in Fig 3a, revealing the dependence of storage modulus (G’) (Fig 3ai) and loss modulus (G”) (Fig 3aii) upon the frequency. G’ measures the deformation energy stored during shear stress, i.e. the material stiffness, while G” measure the dissipated energy, *i*.*e*. the flow or liquid-like response [50]. In all tested periods, G´ was higher than the G”, showing that the produced SF hydrogels are viscoelastic solids, both in a main amorphous conformation (transparent) and in a β-sheet crystalline form (opaque) (Fig 3d). G’ and G” were almost independent on the oscillation frequency, but for higher values of frequency (>1 Hz) it shows a typical plastic flow behavior with G’ and G” increasing with frequency. Moreover, the G’ increases substantially from day 3 to day 7, corresponding to the conformation transition period of hydrogels (Fig 3d). In the first 3 days is suggested a network-like structure converted in a liquid crystal-like structure from day 7 to day 14 of analysis [51]. The damping factor or loss factor (tan δ) was measured by determining the ratio G″/G′ (Table 1). An increase of tan δ was observed over time (from day 1 until day 14), as well as, that the damping factor was lower than 1 in all tested periods (higher storage modulus—G’), characterizing the typical behavior of elastic solids [51]. Moreover, a substantial increase of the damping factor was determined from day 3 to day 7, consistent to the G’ measurements (Fig 3ai). SF/HRP/H_2_O_2_ mixture was subjected to two different oscillatory experiments, temperature (Fig 3bi) and time (Fig 3bii) sweep curves, before gelation. In both cases, a gel-like response was observed in which G’ was always higher than G”. An abrupt increase of G’ was observed up to 27 °C and 120 s, indicating an increase of samples stiffness associated to the formation of a hydrogel network [18]. After a stagnation at intermediate times and on the range of temperature from 30°C to 40°C, a slight increase of G’ and G” was observed at the end of the curves (starting at 40°C and 2900 s), indicating that at this point the SF hydrogels were completely formed and the temperature or time did not significantly alter the final mechanical properties of the hydrogels. The prepared SF/HRP/H_2_O_2_ mixture was studied through rotational experiments (Fig 3c). Before gelation, SF/HRP/H_2_O_2_ mixture showed an average shear viscosity of 4.7 ± 0.37 Pa.s (Fig 3ci), as confirmed by the slope of the linear trend line (Fig 3cii). The shear viscosity values decreased with increasing shear rate (0.1 s^-1^ to 1 s^-1^), suggesting the shear-thinning fluidic behavior of the SF/HRP/H_2_O_2_ mixture. At higher shear rate values (1 s^-1^ to 100 s^-1^) a Newtonian behavior was observed represented by a constant shear viscosity [52].

**Table 1:** Damping factor of SF hydrogels after incubation in PBS at 37°C for 1, 3, 7, 10 and 14 days.

Time (day)	Damping factor
1	0.0121 ± 0.0004
3	0.0208 ± 0.0042
7	0.0941 ± 0.0048
10	0.1039 ± 0.0074
14	0.1078 ± 0.0017

### Assessment of the programed cell death in U251 cell-laden SF hydrogels and live conformational changes

**Fig 4:**
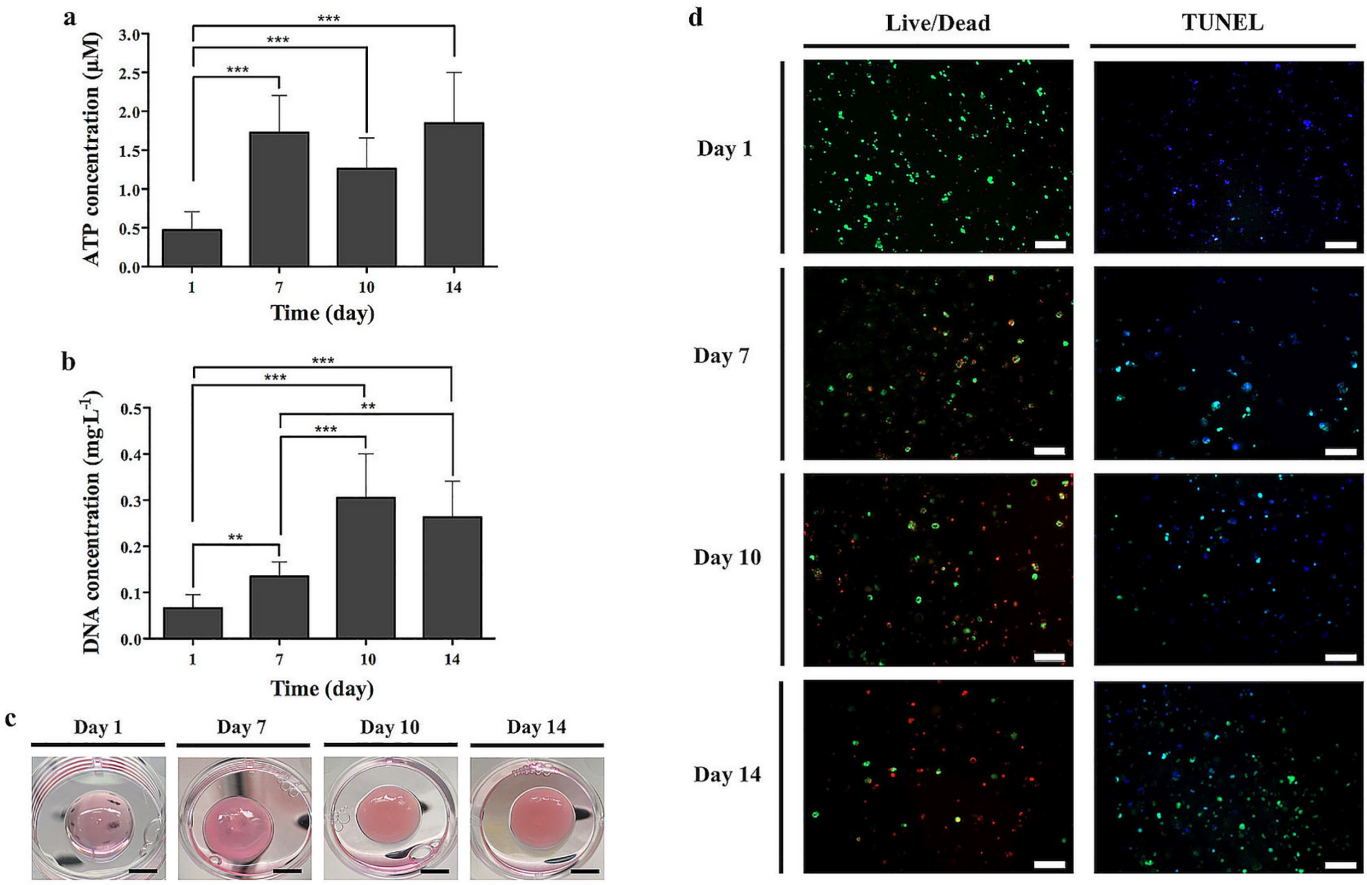
U251 cell-laden SF hydrogels cultured for 1, 7, 10 and 14 days. Cell viability and proliferation analyzed by (a) ATP assay and (b) DNA quantification, respectively. (c) Macroscopic images of the U251 cell-laden SF hydrogels (scale bar, 5 mm). (d) Live/Dead staining and fluorescence TUNEL assay of the U251 cell-laden SF hydrogels (scale bar, 200 μm). All the raw numerical data were provided in S1 and S2 Tables. **p < 0.01, ***p < 0.001.

Due to the conformational transitions observed on the SF hydrogels after short incubation periods, the developed hydrogels were used for U251 cell line encapsulation to evaluate the effects of the conformational changes on cell behavior. The ATP evaluation (Fig 4a) showed that the metabolic activity of the encapsulated cells significantly improved from day 1, as compared to the remaining culture periods (***p < 0.001). Nevertheless, after 10 days of culture a non-significant decrease in cellular metabolic activity was verified, as compared to day 7, corresponding to the conformation transition state of the SF hydrogels, from amorphous (transparent hydrogels) to crystalline β-sheet (opaque hydrogels) (Fig 4c). A non-significant increase of metabolically active cells was observed from day 10 to day 14. This cell behavior may have been induced as stress response to counter the effects of the conformational transition on SF hydrogels. Moreover, in contrast to normal differentiated cells most cancer cells respond differently to generate the energy needed for cellular processes, which affects the ATP production by cells [53]. From DNA quantification (Fig 4b), it was observed a significant increase of cell proliferation from day 1 to day 10 (**p < 0.01: from day 1 to day 7; ***p < 0.001: from day 1 to day 10 and from day 7 to day 10). However, at day 14 a slight decrease in cell proliferation was observed as compared to day 10, even if it has been significantly superior as compared to day 1 (***p < 0.001) and day 7 (**p < 0.01). As shown in Fig 4d, live/dead staining showed that the cell-laden SF hydrogels were capable support cell viability during the first 24 hours of culturing. At day 7 and day 10 the amount of dead cells was very similar to the living ones, and a large amount of dead cells were observed after 14 days of culturing. Cell apoptosis detected through TUNEL assay (Fig 4d) also revealed that no apoptosis was visualized at day 1. However, over the 14 days of culturing a substantial increase of apoptotic cells were detected at the cell-laden hydrogels, presenting at day 14 a very similar pattern to that observed on the positive control samples (S3a Fig). From Figs 1b and 4c it was observed that, both the un-laden and cell-laden SF hydrogels, respectively, maintained a transparent morphology until day 7, where opacity started to be noticed. After 10 days of incubation, hydrogels become completely opaque. The hydrogels TEM, ThT and OPT images (Fig 1c–1e; S1–S5 Movies), showed an increase of SF nanofibrils aggregates after 7 days of incubation and the rheological analysis also showed a substantial increase of hydrogels stiffness from day 7 (Fig 3ai). These results indicate that the unquestionable conformational transition of SF hydrogels to a dominant β-sheet crystalline conformation affected cell proliferation and viability, increasing cell apoptosis.

**Fig 5:**
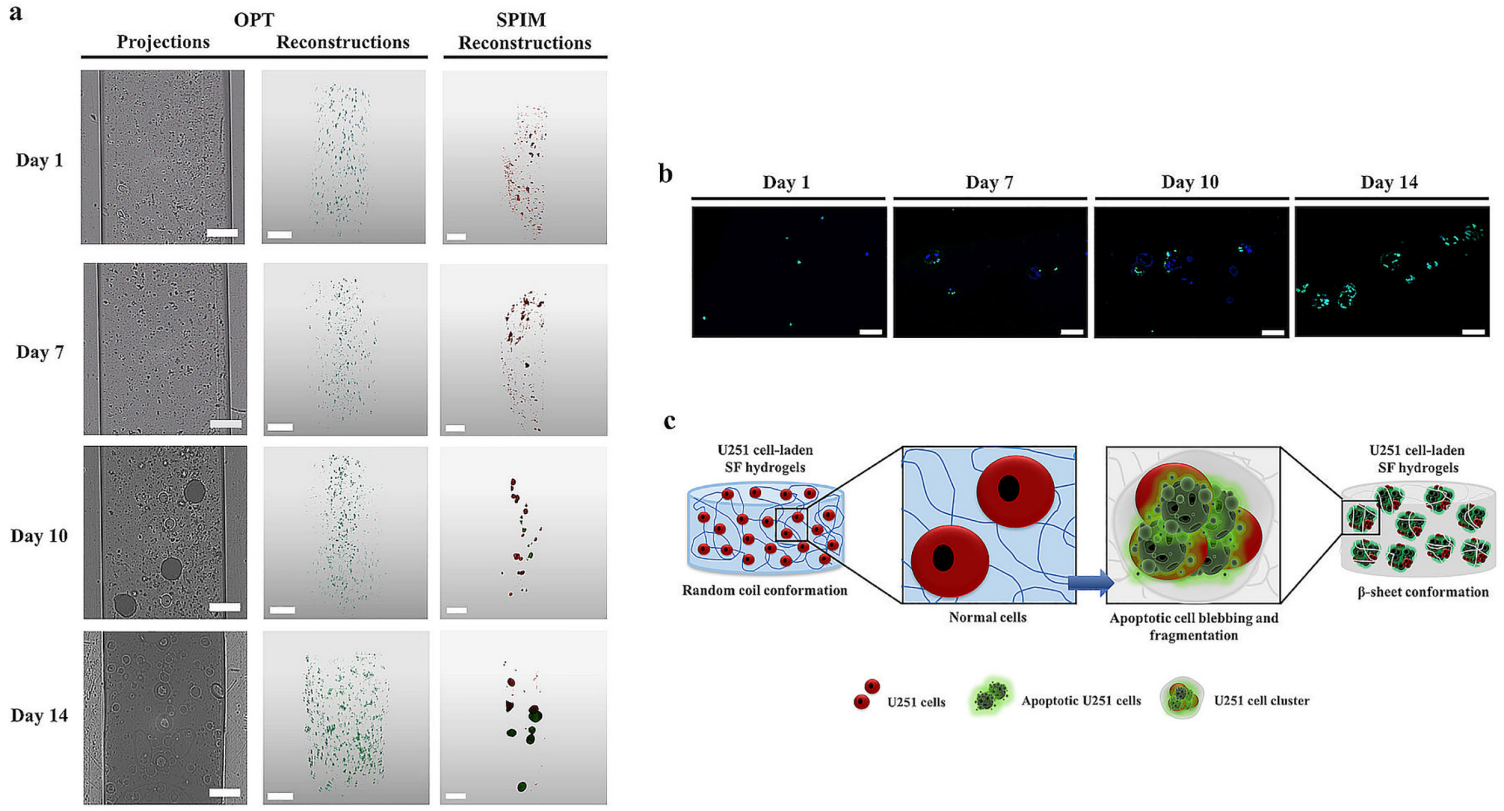
U251 cell-laden SF hydrogels cultured for 1, 7, 10 and 14 days. (a) OPT projections (scale bar, 400 μm), OPT reconstructions (scale bar scale bar, 500 μm) and SPIM reconstructions (scale bar, 200 μm) of the U251 cell-laden SF hydrogels. Movies from OPT projections, OPT reconstructions and SPIM reconstructions of the cell-laden SF hydrogels are available in S6–S17 Movies. (b) Fluorescence TUNEL assay of the sections from the U251 cell-laden SF hydrogels for 1, 7, 10 and 14 days (scale bar, 50 μm). (c) Schematic illustration of the morphological changes of U251 cells during apoptosis induced by the conformational transitions of the cell-laden SF hydrogels.

The *in vitro* conformation transition behavior of the U251 cell-laden SF hydrogels was also evaluated by OPT analysis and selective plane illumination microscopy (SPIM) (Fig 5a; S6–S17 Movies). These imaging techniques allowed to create multi-scale 3D rendered images to evaluate cell distribution within the hydrogels, while being minimally invasive at the same time. OPT projections (Fig 5a; S6–S9 Movies) showed that from day 1 to day 10, U251 cells were well distributed within the hydrogels presenting a main amorphous conformation (transparent morphology) on the first 7 days of culture. At day 10 some changes were observed in the cell-laden hydrogels morphology due to the presence of some opaque depots characteristic of a β-sheet conformation transition. These opaque depots were converted in the main structure of the U251 cell-laden SF hydrogels after 14 days of culture, showing the complete transition of hydrogels conformation and confirming the previous OPT observations (Fig 1e; S1–S5 Movies). Nevertheless, cell distribution behavior after 14 days of culture was only possible to evaluate through OPT reconstructions (Fig 5a; S10–S13 Movies), showing that cells were organized in clusters. SPIM reconstructions (Fig 5a; S14–S17 Movies), allowed not only to achieve high resolution 3D images of the cell-laden SF hydrogels using high penetration depths, as imaged and tracked the cells within the hydrogels by fluorescence labeling. At day 1 and day 7, U251 cells presented a very similar distribution within the hydrogels to that observed on OPT projections (Fig 5a; S6–S9 Movies) and reconstructions (Fig 5a; S10–S13 Movies). At day 10, cells were already organized in clusters and this effect was even more pronounced after 14 days of cell encapsulation. The sections from the U251 cell-laden hydrogels analyzed by TUNEL assay (Fig 5b) also showed an increase of cell clusters over the culture period accompanied by a substantial increase of apoptotic cells on day 14, suggesting that the transition from random coil (amorphous) to a β-sheet (crystalline) (Fig 4c) induced formation of U251 cell clusters that undergo a programmed cell death (Fig 5c; S2c Fig).

## Discussion

In this study, we have investigated enzymatically crosslinked SF hydrogels mediated by a HRP/H_2_O_2_ complex. These hydrogels were produced in a main random coil conformation, which allow them to obtain physical, mechanical and biological properties completely distinct to those reached by the SF hydrogels formed with a β-sheet conformation [17, 18], or even to those induced by electrical stimuli in main random coil conformation [54]. The high concentration of SF (16 wt%) allowed us to obtain fast-formed hydrogels (15–20 minutes of gelling time) suitable for injectable systems [55]. The previous electrically induced SF hydrogels, were prepared from low concentrated SF, which imply longer gelation times and low mechanical properties [54]. As observed in a previous study by our group [20], the proposed SF hydrogels can be customized to yield a wide range gelling times and mechanical properties only by changing the concentration of SF and the HRP/H_2_O_2_ ratio, due to the tyrosine groups available in the crosslinking system [22]. The crosslinking system herein proposed, involved not only a highly-concentrated SF solution (16 wt%), as an increased concentration of enzyme (HRP) was applied to yield faster gelation times. Moreover, the proposed highly concentrated SF hydrogels have shown high water content and swelling ability, both in the amorphous state and after β-sheet inducement, which is very important to maintain hydrogels integrity and better simulate the physiological environment [21].

The proposed rapidly responsive SF hydrogels have been previously observed to present a transparent appearance during the amorphous conformation state that gradually changes to an opaque morphology when the hydrogels are naturally converted in a β-sheet conformation [20]. This was another interesting characteristic of our hydrogels that presented significant morphological changes after 7 days of hydrogels formation, indicative of a conformational transition of SF to β-sheet crystalline form, as confirmed under TEM, ThT staining and OPT analysis (Fig 1c–1e; S1–S5 Movies). These results also suggest that the applied enzymatic crosslinking on the semi-crystalline or amorphous tyrosine groups of SF may not affect the β-sheet inducement of the protein [22]. In a previous study, Ryu *et al*. [56] proposed a different approach for producing SF-PEG hybrid hydrogels, where the SF phase was naturally converted to a β-sheet conformation after 5 days of hydrogels formation. These hydrogels were formed by an initial functionalization step of SF in order to allow the photo-polymerization reaction and hydrogels formation. The post-gelation step of SF was then conducted by the natural β-sheet structural transition within the hydrogel network. Although most of the reported literature recognize that the formation of transparent SF hydrogels is associated with a random-coil conformation and opaque hydrogels present a main β-sheet conformation, it has been also reported the development of transparent SF hydrogels formed by reaction with polar reagents that resulted in an amorphous-to-crystalline conformational change [57].

The chemical characterization of the SF hydrogels (Fig 2a, 2b and 2e) was essential to confirm the multi-scale morphological observations and in which state of hydrogels formation the conformational transition takes place, showing that until day 7 the developed SF hydrogels presented the typical low intensity peak positions of a random coil conformation. After 14 days of hydrogels formation, high intensity absorption peaks were detected resulting from an increase of crystallinity and conversion to a dominant β-sheet conformation. Thus, understanding the structure-property relation of protein-based biomaterials can open for new possibilities of developing high-level 3D biomaterials impossible to obtain from other polymeric materials [58]. Mechanical properties constitute one of the main issues of biomaterials design for TE applications. In the specific case of hydrogels, it can be hard to produce with sufficient mechanical strength to support tissues like cartilage or subchondral bone [59]. Moreover, in most cases high elasticity is also required, which hindered its production for the desired applications. In this study, SF hydrogels presented a viscoelastic solid behavior, both in a random coil conformation or in a β-sheet opaque form [51]. Even the materials stiffness has suffered a substantial increase during the conformational transition, they still maintained the typical behavior of elastic solids, which is not the normal behavior of SF hydrogels formed in a β-sheet conformation with high stiffness but lack of elasticity [17]. The crosslinking process induced by the enzymatic complex of HRP/H_2_O_2_, may had a direct influence on these results [8]. As previously observed, higher contents of peroxidase and hydrogen peroxide in the crosslinking system, equivalent to those used in the present system (HRP/silk: 0.52‰; H_2_O_2_/silk: 1.45‰), can induce higher amount of oxidized tyrosine groups and an enhanced crosslinking degree that resulted in improved mechanical properties of the hydrogels [20]. Moreover, in both studies it was observed that regardless of the enzymatic ratio, in the amorphous state SF hydrogels presented a constant elasticity with increasing frequency and strain sweep. We were also able to distinguish the gelling point of the SF/HRP/H_2_O_2_ mixture by the substantial increase of samples stiffness at specific temperature and time sweep [18] and the viscoelastic behavior was also determined to evaluate the resistance to flow, which is an important concern of injectable systems [4, 51]. Polymers are known for being non-Newtonian fluid and most of them exhibit shear-thinning behavior in which the molecules are oriented along the flow direction, as observed in our results. At high shear rate, the Newtonian behavior was observed due to the molecules disorder as a result of shearing [52].

In the hydrogel-based 3D cell cultures, the seeding of cells is usually done by suspending cells in the fluidic gel precursor solution, in order to obtain the cells embedded inside the gel after gelation [60]. This is one of the major limitations of the SF hydrogels reported in the literature [17, 18]. As above mentioned, the usually applied methods for preparing SF hydrogels involve the β-sheet conformational transition of SF during the sol-gel transition, which in some cases may involve long gelation times [61]. On the other side, physical and chemical treatments proposed to shorten the SF gelation time, may also have provided useful timeframes for successful cell encapsulation [17, 18]. For example, the ultrasonication treatment of SF have shown to decrease the gelation time of SF hydrogels up to 2 hours, at the same time that a successful cell incorporation after sonication and before the rapid gelation process was observed. These hydrogels sustained cell function, proliferation and survival up to 21 days of culture [17]. The same protocol was used in a different study [62], allowing the co-encapsulation of pancreatic iselets with mesenchymal stem cells (MSCs) and ECM proteins within vortex-induced SF hydrogels. In the present study, the SF hydrogels were prepared at physiological conditions (peroxidase mediated crosslinking) and in a main random coil conformation, allowing for cell encapsulation, viability and proliferation up to 10 days of culture, corresponding to the period of SF amorphous-to-crystalline conformational transition (Figs 1 and 2; S1–S5 Movies). The complete transition to a dominant β-sheet conformation was verified at day 14, accompanied by a substantial increase of cell apoptosis. In previous studies [31, 63], hydrogel matrices presented superior physiological properties as *in vitro* platforms for cancer cells encapsulation and proliferation, where cells were encouraged to grow as tumor-like clusters in 3D formation. Moreover, the authors also observed that the oxygen and nutrients diffusion limitations within the hydrogel matrices resulted in cellular competition for the available nutrients, growing levels of intra-cellular hypoxia, and as consequence in the development of necrosis in the core of the *in vitro* bioengineered tumors [63]. Our suggestion is that the opacity and crystallinity induced by the β-sheet conformation may have conditioned the oxygen and nutrients diffusion within the cell-laden hydrogels, forcing the cell cluster formation. At some point, cancer cells were not able to adjust to those deficiencies ceding to a cell death by apoptosis (Fig 5c). It is important to reinforce that this cell behavior may be beneficial to mimic the tumor microenvironment in 3D cancer models research. The relation of a cell death induced by the β-sheet conformation of SF protein can also be raised, especially since it has been reported in the literature that fibrillar β-amyloid peptides may have cytotoxic properties [64]. Nevertheless, different SF-based structures in a β-sheet form, have proved to be able of support cell viability, proliferation and differentiation, as well as, *in vivo* biocompatibility [16]. From the OPT and SPIM reconstructions (Fig 5a; S10–S17 Movies), we were able to observe the typical behavior of cells in growing tumors, with an increase of U251 cell clusters over the culture period, especially after 14 days of culture. This cell behavior may be beneficial to mimic the tumor microenvironment in 3D cancer models research. In fact, bioengineered 3D hydrogels have already shown to induce cancer cells clusters formation in a new 3D culture concept used to assess the cell-matrix interactions implied in carcinogenesis [65]. From the cell-laden hydrogels TUNEL sections (Fig 5b) it was possible to closely observe that at day 14 most of these clustered cells were apoptotic, confirming that the β-sheet crystalline domain of SF hydrogels may not only affect cell distribution but also induce programmed cell death (Fig 5c), which reinforces the potential use of these hydrogels as biomimetic matrices for studying the programmed tumor cells death.

## Conclusions

The present work demonstrates the formation of stimuli-responsive enzymatically crosslinked SF hydrogels that undergo a spontaneous conformational transition from random coil to β-sheet at physiological conditions. These hydrogels were highly resistant and presented appropriate mechanical properties to be used as injectable systems or 3D artificial matrices. They were successfully applied for cell encapsulation, showing a significant increase of U251 cells proliferation and metabolic activity in the transparent amorphous state. The spontaneous random coil-to-β-sheet conformational transition of the SF hydrogels affected the viability of cells and induced apoptosis, envisioning their use as a generic injectable system to guide cancer cells behavior and suppressing tumor progression. Furthermore, the stimuli-responsiveness and cell-loading ability of these *in situ* forming SF hydrogels, provide new insights for using these hydrogels as potential orthotopic 3D cancer models and for studying the 3D microenvironment of tumor cells.

## Supporting information

S1 Fig Schematic illustration of redox-responsive enzymatically crosslinked SF hydrogels. (a) Oxidation-reduction reaction between HRP and H_2_O_2_ transformed the tyrosine groups of SF and induced hydrogels formation. (b) Rapidly responsive sol-gel transition combining HRP and H_2_O_2_ at physiological conditions (pH 7.4 and 37°C).
(TIF)

S2 Fig Schematic illustration of *in situ* fast-formed enzymatically crosslinked SF hydrogels and *in vitro* response of U251 cell-laden SF hydrogels. (a) Peroxidase mediated crosslinking method using HRP and H_2_O_2_ at physiological conditions (pH 7.4 and 37°C), reacting with the tyrosine groups of the SF protein. (b) U251 cells encapsulation within the newly formed random coil SF hydrogels. (c) U251 cell-laden SF hydrogels converted into a crystalline β-sheet conformation showing U251 cell clusters organization and U251 cell death by apoptosis.
(TIF)

S3 Fig Positive control for fluorescence TUNEL assay. (a) U251 cell-laden SF hydrogels (scale bar, 200 μm) and (b) sections from the U251 cell-laden SF hydrogels (scale bar, 50 μm).
(TIF)

S1 Table Data points behind the mean values obtained from the ATP quantification assay. (DOCX)

S2 Table Data points behind the mean values obtained from the DNA quantification assay. (DOCX)

S1 Movie OPT reconstruction of the SF hydrogels after incubation in PBS at 37°C for 1 day. (MPG)

S2 Movie OPT reconstruction of the SF hydrogels after incubation in PBS at 37°C for 3 days. (MPG)

S3 Movie OPT reconstruction of the SF hydrogels after incubation in PBS at 37°C for 7 days. (MPG)

S4 Movie OPT reconstruction of the SF hydrogels after incubation in PBS at 37°C for 10 days. (MPG)

S5 Movie OPT reconstruction of the SF hydrogels after incubation in PBS at 37°C for 14 days. (MPG)

S6 Movie OPT projection of the U251 cell-laden SF hydrogels cultured for 1 day. (MPG)

S7 Movie OPT projection of the U251 cell-laden SF hydrogels cultured for 7 days. (MPG)

S8 Movie OPT projection of the U251 cell-laden SF hydrogels cultured for 10 days. (MPG)

S9 Movie OPT projection of the U251 cell-laden SF hydrogels cultured for 14 days. (MPG)

S10 Movie OPT reconstruction of the U251 cell-laden SF hydrogels cultured for 1 day. (MPG)

S11 Movie OPT reconstruction of the U251 cell-laden SF hydrogels cultured for 7 days. (MPG)

S12 Movie OPT reconstruction of the U251 cell-laden SF hydrogels cultured for 10 days. (MPG)

S13 Movie OPT reconstruction of the U251 cell-laden SF hydrogels cultured for 14 days. (MPG)

S14 Movie SPIM reconstruction of the U251 cell-laden SF hydrogels cultured for 1 day. (MPG)

S15 Movie SPIM reconstruction of the U251 cell-laden SF hydrogels cultured for 7 days. (MPG)

S16 Movie SPIM reconstruction of the U251 cell-laden SF hydrogels cultured for 10 days. (MPG)

S17 Movie SPIM reconstruction of the U251 cell-laden SF hydrogels cultured for 14 days. (MPG)
