# Supplementary figures and images for: Rapidly responsive silk fibroin hydrogels as an artificial matrix for the programmed tumor cells death
Source: PLoS One. 2018 Apr 4;13(4):e0194441. doi: 10.1371/journal.pone.0194441 (PMC5884513; doi:10.1371/journal.pone.0194441)

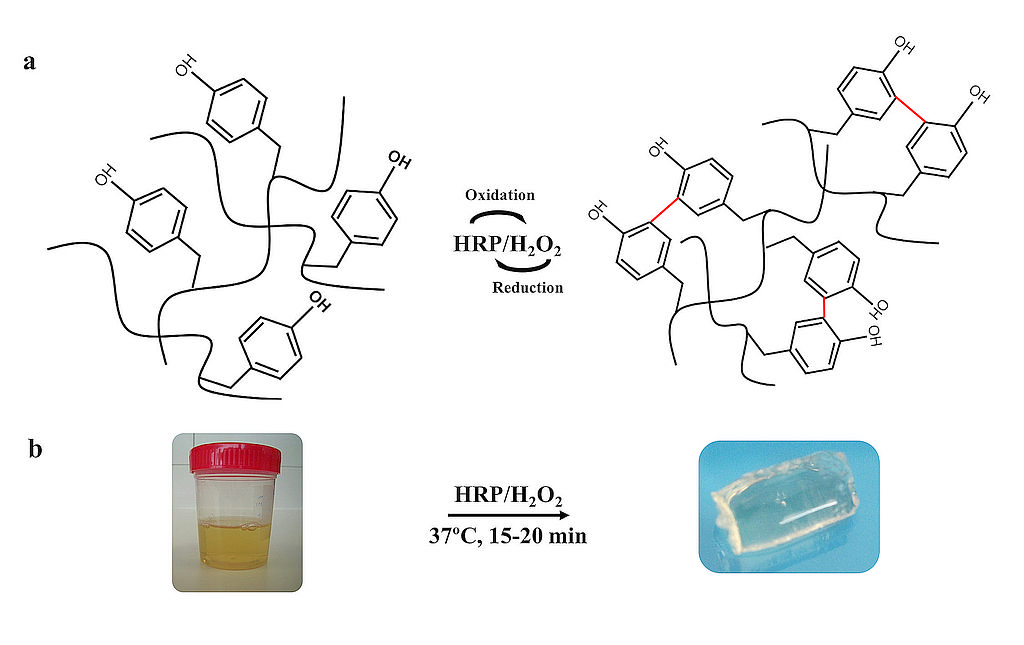

Supplement: S1 Fig — (a) Oxidation-reduction reaction between HRP and H2O2 transformed the tyrosine groups of SF and induced hydrogels formation. (b) Rapidly responsive sol-gel transition combining HRP and H2O2 at physiological conditions (pH 7.4 and 37°C). (TIF) [file pone.0194441.s001.tif]

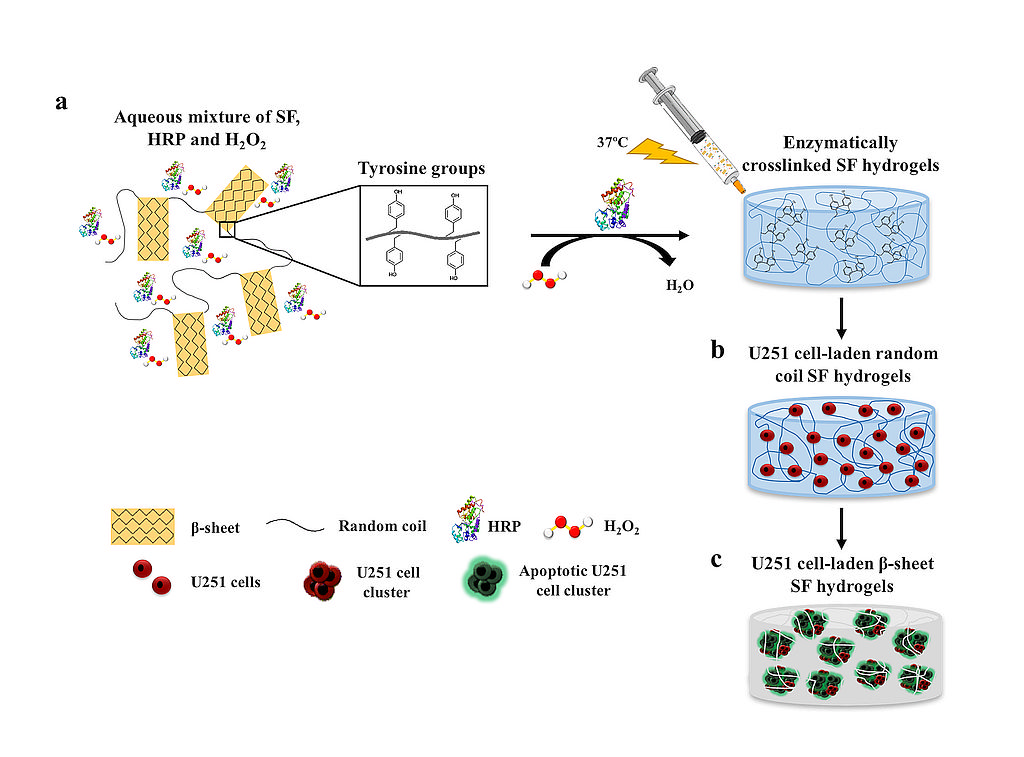

Supplement: S2 Fig — (a) Peroxidase mediated crosslinking method using HRP and H2O2 at physiological conditions (pH 7.4 and 37°C), reacting with the tyrosine groups of the SF protein. (b) U251 cells encapsulation within the newly formed random coil SF hydrogels. (c) U251 cell-laden SF hydrogels converted into a crystalline β-sheet conformation showing U251 cell clusters organization and U251 cell death by apoptosis. (TIF) [file pone.0194441.s002.tif]

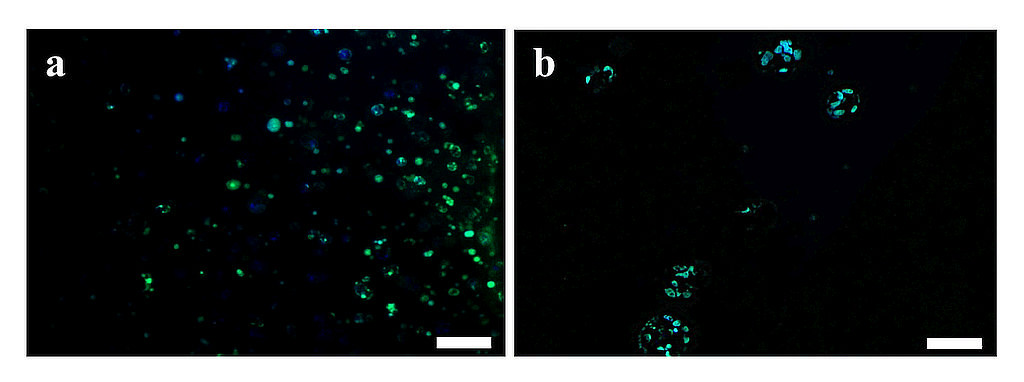

Supplement: S3 Fig — (a) U251 cell-laden SF hydrogels (scale bar, 200 μm) and (b) sections from the U251 cell-laden SF hydrogels (scale bar, 50 μm). (TIF) [file pone.0194441.s003.tif]
