# Supplementary material for: Rapidly responsive silk fibroin hydrogels as an artificial matrix for the programmed tumor cells death
Source: PLoS One. 2018 Apr 4;13(4):e0194441. doi: 10.1371/journal.pone.0194441 (PMC5884513; doi:10.1371/journal.pone.0194441)
Supplement: S1 Table — (DOCX) [file pone.0194441.s004.docx]

| **Day 1** | | **Day 7** | | **Day 10** | | **Day 14** | |
| --- | --- | --- | --- | --- | --- | --- | --- |
| **1^st^ Experiment** | 0.070843332 | **1^st^ Experiment** | 0.990990618 | **1^st^ Experiment** | 0.918352818 | **1^st^ Experiment** | 2.30028981 |
|  | 0.070702457 |  | 1.027615908 |  | 0.918803363 |  | 2.304401326 |
|  | 0.064433527 |  | 0.976224556 |  | 0.935369523 |  | 2.393592367 |
|  | 0.291488538 |  | 1.872180088 |  | 1.376486873 |  | 2.871718381 |
|  | 0.284550453 |  | 1.985841253 |  | 1.414297921 |  | 2.805465271 |
|  | 0.283634766 |  | 1.965857621 |  | 1.416723928 |  | 2.777550242 |
|  | 0.266518474 |  | 1.533549614 |  | 1.341067173 |  | 2.546980761 |
|  | 0.250317868 |  | 1.590192637 |  | 1.303637354 |  | 2.541426608 |
|  | 0.237956102 |  | 1.588010124 |  | 1.320965974 |  | 2.62113231 |
| **2^nd^ Experiment** | 0.553079288 | **2^nd^ Experiment** | 2.749691208 | **2^nd^ Experiment** | 1.799171433 | **2^nd^ Experiment** | 1.256893544 |
|  | 0.548015996 |  | 2.691792684 |  | 1.802430323 |  | 1.252378122 |
|  | 0.558905123 |  | 2.838114929 |  | 1.836456969 |  | 1.269642972 |
|  | 0.857883314 |  | 2.000862898 |  | 1.412588192 |  | 1.346893544 |
|  | 0.873103689 |  | 1.838928198 |  | 1.398627187 |  | 1.562378122 |
|  | 0.880820633 |  | 1.920026179 |  | 1.403819372 |  | 1.509642972 |
|  | 0.503147917 |  | 1.500288192 |  | 1.607739201 |  | 2.209392012 |
|  | 0.507326657 |  | 1.560212301 |  | 1.652293812 |  | 2.212993812 |
|  | 0.531453544 |  | 1.606638911 |  | 1.709991728 |  | 2.197736183 |
| **3^rd^ Experiment** | 0.413008258 | **3^rd^ Experiment** | 1.875369922 | **3^rd^ Experiment** | 1.345962408 | **3^rd^ Experiment** | 1.854833394 |
|  | 0.421140381 |  | 1.861481918 |  | 1.369328856 |  | 1.826168266 |
|  | 0.422302113 |  | 1.921143 |  | 1.336657805 |  | 1.860754686 |
|  | 0.566576659 |  | 1.559003737 |  | 1.067139126 |  | 1.37605895 |
|  | 0.571568966 |  | 1.619715975 |  | 1.046920853 |  | 1.213208227 |
|  | 0.559135295 |  | 1.660902189 |  | 1.001622126 |  | 1.313262885 |
|  | 0.731291406 |  | 1.296947294 |  | 0.419909519 |  | 0.787118102 |
|  | 0.709940657 |  | 1.256111465 |  | 0.442121636 |  | 0.826836309 |
|  | 0.726173506 |  | 1.294080504 |  | 0.455483886 |  | 0.831117859 |
