# Supplementary material for: Rapidly responsive silk fibroin hydrogels as an artificial matrix for the programmed tumor cells death
Source: PLoS One. 2018 Apr 4;13(4):e0194441. doi: 10.1371/journal.pone.0194441 (PMC5884513; doi:10.1371/journal.pone.0194441)
Supplement: S2 Table — (DOCX) [file pone.0194441.s005.docx]

| **Day 1** | | **Day 7** | | **Day 10** | | **Day 14** | |
| --- | --- | --- | --- | --- | --- | --- | --- |
| **1^st^ Experiment** | 0.06124327 | **1^st^ Experiment** | 0.09125673 | **1^st^ Experiment** | 0.402073576 | **1^st^ Experiment** | 0.28524401 |
|  | 0.06049345 |  | 0.083316365 |  | 0.393674065 |  | 0.302806625 |
|  | 0.068892961 |  | 0.119968778 |  | 0.405891536 |  | 0.289825562 |
|  | 0.114708478 |  | 0.206254666 |  | 0.36007602 |  | 0.116490192 |
|  | 0.123107989 |  | 0.20778185 |  | 0.373057083 |  | 0.122598928 |
|  | 0.120817213 |  | 0.184874092 |  | 0.452470644 |  | 0.120308152 |
|  | 0.059729858 |  | 0.104696939 |  | 0.337931854 |  | 0.282953234 |
|  | 0.06431141 |  | 0.099351795 |  | 0.339459038 |  | 0.286007602 |
|  | 0.06049345 |  | 0.114623634 |  | 0.326477975 |  | 0.26157266 |
| **2^nd^ Experiment** | 0.037670536 | **2^nd^ Experiment** | 0.135664834 | **2^nd^ Experiment** | 0.391213602 | **2^nd^ Experiment** | 0.315618297 |
|  | 0.046070047 |  | 0.134901242 |  | 0.411066992 |  | 0.393504378 |
|  | 0.048360823 |  | 0.137192018 |  | 0.411066992 |  | 0.406485441 |
|  | 0.087304011 |  | 0.129768313 |  | 0.353682017 |  | 0.326794512 |
|  | 0.094939931 |  | 0.128979954 |  | 0.330025701 |  | 0.353890144 |
|  | 0.118611281 |  | 0.130013458 |  | 0.309986341 |  | 0.337814902 |
|  | 0.036143352 |  | 0.139023819 |  | 0.307357923 |  | 0.299998291 |
|  | 0.028507432 |  | 0.140872839 |  | 0.317728192 |  | 0.275288102 |
|  | 0.025453065 |  | 0.141521929 |  | 0.367929931 |  | 0.302991821 |
| **3^rd^ Experiment** | 0.020871513 | **3^rd^ Experiment** | 0.150554877 | **3^rd^ Experiment** | 0.166463042 | **3^rd^ Experiment** | 0.284437996 |
|  | 0.049124415 |  | 0.142918957 |  | 0.163408674 |  | 0.278329261 |
|  | 0.045306455 |  | 0.141391774 |  | 0.151191203 |  | 0.28596518 |
|  | 0.068214213 |  | 0.104739361 |  | 0.222205254 |  | 0.175244349 |
|  | 0.073559357 |  | 0.110848096 |  | 0.219914478 |  | 0.176771533 |
|  | 0.093412747 |  | 0.09252189 |  | 0.218387294 |  | 0.169135614 |
|  | 0.054469558 |  | 0.154372836 |  | 0.176389737 |  | 0.202389012 |
|  | 0.057523926 |  | 0.155136428 |  | 0.155009163 |  | 0.229757910 |
|  | 0.068214213 |  | 0.158954388 |  | 0.174862553 |  | 0.217752891 |
